# Molecular Characterization of TGF-β Type I Receptor Gene (*Tgfbr1*) in *Chlamys farreri*, and the Association of Allelic Variants with Growth Traits

**DOI:** 10.1371/journal.pone.0051005

**Published:** 2012-11-29

**Authors:** Huihui Guo, Zhenmin Bao, Jiqin Li, Shanshan Lian, Shi Wang, Yan He, Xiaoteng Fu, Lingling Zhang, Xiaoli Hu

**Affiliations:** Key Laboratory of Marine Genetics and Breeding, Ministry of Education, College of Marine Life Sciences, Ocean University of China, Qingdao, China; Temasek Life Sciences Laboratory, Singapore

## Abstract

**Background:**

Scallops are an economically important aquaculture species in Asian countries, and growth-rate improvement is one of the main focuses of scallop breeding. Investigating the genetic regulation of scallop growth could benefit scallop breeding, as such research is currently limited. The transforming growth factor beta (TGF-β) signaling through type I and type II receptors, plays critical roles in regulating cell proliferation and growth, and is thus a plausible candidate growth regulator in scallops.

**Results:**

We cloned and characterized the TGF-β type I receptor (*Tgfbr1*) gene from Zhikong scallops (*Chlamys farreri*). The deduced amino acid sequence contains characteristic residues and exhibits the conserved structure of Tgfbr1 proteins. A high expression level of scallop *Tgfbr1* was detected during early embryonic stages, whereas *Tgfbr1* expression was enriched in the gonad and striated muscle in adults. A single nucleotide polymorphism (SNP, c. 1815C>T) in the 3′ UTR was identified. Scallops with genotype TT had higher growth traits values than those with genotype CC or CT in a full-sib family, and significant differences were found between genotypes CC and TT for shell length, shell height, and striated muscle weight. An expression analysis detected significantly more *Tgfbr1* transcripts in the striated muscle of scallops with genotype CC compared to those with genotype TT or CT. Further evaluation in a population also revealed higher striated muscle weight in scallops with genotype TT than those with the other two genotypes. The inverse correlation between striated muscle mass and *Tgfbr1* expression is consistent with TGF-β signaling having a negative effect on cell growth.

**Conclusion:**

The scallop *Tgfbr1* gene was cloned and characterized, and an SNP potentially associated with both scallop growth and *Tgfbr1* expression was identified. Our results suggest the negative regulation of *Tgfbr1* in scallop growth and provide a candidate marker for Zhikong scallop breeding.

## Introduction

Scallops are widely distributed throughout the world and represent economically important aquaculture species in Asian countries [1]; in China, scallop culture contributes over 1 million metric tons of production per year [2]. The selective breeding of scallops with rapid growth rates has been a primary focus of scallop farming, and genetic breeding programs aiming to improve growth rates have recently been launched. Therefore, the identification of genes with putative functions in growth regulation or genetic markers associated with growth in scallops could provide useful information for these targeted breeding programs. However, such research is quite limited at present.

The transforming growth factor beta (TGF-β) superfamily, which includes activins, bone morphogenetic proteins (BMPs), TGF-βs, growth and differentiation factors (GDFs), and other related factors, regulates diverse cellular processes, such as proliferation, differentiation, growth, adhesion, and apoptosis, in species ranging from flies and nematodes to mammals [3], [4]. These secreted growth factors transduce signals through an evolutionarily conserved mechanism involving type I and type II serine/threonine kinase receptors on the cell surface [5], [6]. In most cases, TGF-β ligand dimers initiate the signaling cascade by binding to two type II receptors, which then recruit two type I receptors, and phosphorylate their characteristic GS (glycine-serine tandem repeat) motifs, to form an active receptor signaling complex comprising both types of receptors and the ligand dimer. The activated type I receptors propagate the signal from the cell surface to the cytoplasm, through the transphosphorylation of Smad proteins which then enter the nucleus to regulate the transcription of target genes [7]. The activation of the type I receptors is regarded as the central event in the generation of signals by TGF-βs [6], [8], [9].

In general, TGF-β signaling has a negative effect on cell growth [4], making this pathway an attractive candidate for studies on controlling human cancer or regulating animal growth [10], [11]. As the major type I receptor for TGF-β ligands, such as TGF-β1, TGF-β2, TGF-β3, and GDF8 (myostatin) in mammals [11], [12], the TGF-β type I receptor (Tgfbr1) has been of great interest to researchers, and associations between *Tgfbr1* allelic variations and gene expression or diseases have been explored in humans, revealing several causative loci [13]–[16]. Porcine *Tgfbr1* has been studied extensively, as it falls within a quantitative trait locus (QTL)-rich region to which many growth and reproductive traits have been mapped [17]–[19], and single nucleotide polymorphisms (SNPs) significantly associated with porcine growth rates, including average daily gains during several growing stages, and reproductive traits, including gestation length and number of corpora lutea, were found in porcine *Tgfbr1*, making this gene more valuable in livestock breeding [20].

Several genes encoding TGF-β ligands, type I and II receptors, and Smads have been identified in oysters [21]–[28], suggesting that a similar TGF-β pathway exists in bivalves. In scallops, genes encoding the TGF-β ligand, *myostatin*, were characterized, and SNPs associated with scallop growth traits were identified [29]–[32], implying the function of TGF-β signaling in bivalve growth regulation. However, no scallop TGF-β receptors have been cloned or characterized to date. To better understand how this pathway functions in the scallop, we cloned a TGF-β type I receptor gene, *Tgfbr1*, from the Zhikong scallop (*Chlamys farreri*), which is widely farmed along the northern coast of China, and determined its expression levels during embryonic development and in adult tissues. We identified a single SNP (c. 1815C>T) and evaluated its association with both growth traits and gene expression in striated muscle (the main component of the adductor muscle). Our results suggest the negative regulation of *Tgfbr1* in scallop growth. This SNP might be involved in the regulation of *Tgfbr1* expression in the Zhikong scallop, and could potentially serve as a marker for selective breeding programs for scallop aimed at improving production.

## Materials and Methods

### Ethics Statement

The scallops used in this study were collected from local scallop farms under a permit from the local government. All of the scallop handling was conducted in accordance with the guidelines and regulations established by the Ocean University of China and the local government.

### Scallop Tissues, Embryos and Larvae Collection

For *Tgfbr1* gene cloning, gene expression and SNP analysis, 48 two-year-old offspring from one full-sib Zhikong scallop family were randomly collected in November 2010 from the Xunshan hatchery in Shandong, China. Then 196 two-year-old individuals from a Zhikong scallop population which were collected from Qingdao coast at their larva stage and then cultured in the same cage at a scallop farm of Shazikou, Shandong, China, were randomly selected in December 2011, which were used for further SNP and growth traits association analysis. At the sampling time for the family and population, the male and female gonads cannot be discriminated. The growth traits, including the shell length, shell height, body weight, soft tissue weight and striated muscle weight, were measured for each scallop. Their mantles, gills, gonads, kidneys, striated muscles and digestive glands were dissected, immediately frozen in liquid nitrogen and stored at −80°C. Embryos and larvae, including newly fertilized eggs, 4-cell stage embryos, blastulae, gastrulae, trochophore larvae, D-shaped larvae and veliger larvae were collected in May 2012 and preserved at −80°C.

### RNA Isolation and cDNA Synthesis

Total RNA was isolated from embryos/larvae and tissues of Zhikong scallops using traditional RNA isolation methods [33] and then digested with DNase I (TaKaRa, Shiga, Japan). The first-strand cDNA was synthesized according to the manufacturer’s instruction of M-MLV Reverse Transcriptase (Promega, WI, USA) in a 25-µL volume using 2 µg DNase I-treated total RNA as template and 0.8 µM Oligo (dT)_18_ (TaKaRa) as primer (Table 1). A control reaction without reverse transcriptase was performed to preclude any DNA contamination. The reaction was performed at 42°C for 90 min, terminated by heating at 95°C for 5 min. The cDNA was diluted to 1∶10 and stored at −20°C.

**Table 1:** Sequences of the primers and probes used in this study.

Name (Location^1^)	Sequence (5′ to 3′)^2^	Orientation	Length (bp)^3^
Tgfbr1-f1 (847 to 873)	GAYAAYAARGAYAAYGGIACITGG	Sense	359
Tgfbr1-r1 (1183 to 1205)	GGIGCCATRTAICKYTTIGTICC	Antisense	
Tgfbr1-f2 (929 to 955)	TCAACCGTACTGTGGTCAGCATCTCCG	Sense	91
Tgfbr1-r2 (993 to 1019)	CCAATGATCTCCATGTGGAGGTGGGCG	Antisense	
Tgfbr1-f3 (219 to 242)	GATCTATATTCGAGTCCAAGAAGG	Sense	1625
Tgfbr1-r3 (1821 to 1843)	CATAAAGCTATTCATGTCTTCGG	Antisense	
Tgfbr1-f4 (1760 to 1784)	GAGGTGTCAGACTGATTTTCAGGAG	Sense	101
Tgfbr1-r4 (1836 to 1860)	ATGTCACAAAGGAAATTCATAAAGC	Antisense	
Tgfbr1-Pb (1803 to 1827)	TTGTAAGGAAGT***T***GGATACCGAACT	Probe	
Tgfbr1-f5 (335to 355)	ATCAACACCTGAGACCGACCT	Sense	168
Tgfbr1-r5 (480 to 502)	GGCACTGATGGTAGGCAAAGAAC	Antisense	
Actin-f	CAATCTACGAAGGTTATGCC	Sense	186
Actin-r	CCTGTTCAAAGTCAAGTGC	Antisense	

1 Location represents the position relative to the scallop *Tgfbr1* translation start codon.

2 The bold and italics letter in the probe sequence indicates the position of the SNP.

3 The size of the amplicon.

### Cloning of the Full-length *Tgfbr1* cDNA of Zhikong Scallop

Two degenerate primers, Tgfbr1-f1 and Tgfbr1-r1, corresponding to the conserved amino acid sequences, DNKDNGTW and GTKRYMAP (Table 1), respectively, were used to amplify *Tgfbr1* cDNA fragments from the striated muscle of Zhikong scallop. The PCR was performed in a 50-µL volume containing 1× PCR buffer, 2 mM of MgCl_2_, 2.5 U *Taq* DNA polymerase (TaKaRa, Shiga, Japan), 0.2 mM of each dNTP (Invitrogen, CA, USA), 0.2 µM of each degenerate primer and 2.5 µL diluted cDNA. The amplification was performed as follows: 95°C for 5 min; 30 cycles of 95°C for 30 s, 52°C for 30 s and 72°C for 30 s; and a final extension at 72°C for 5 min. The PCR products were purified and sequenced by Bioasia Biotechnology (Shanghai, China), and a 358 bp cDNA sequence was obtained. To get the full-length cDNA, 3′ and 5′ rapid amplifications of cDNA ends (RACE) were performed using a SMART™ RACE cDNA Amplification Kit (Clontech, CA, USA) according to the manufacturer’s instruction. The specific primers, Tgfbr1-f2 and Tgfbr1-r2, were designed for the 3′ and 5′ RACE, respectively, based on the sequence of the initial 358 bp gene fragment. The RACE fragments were ligated into the pMD18-T vector (TaKaRa), and six recombinant plasmids with inserts for both 3′ and 5′ RACE were sequenced by Bioasia Biotechnology. The full-length cDNA sequence was determined by piecing together the sequences of the 3′ and 5′ RACE products.

### Sequence Analysis

The sequences were analyzed using the BLAST algorithm at the National Center for Biotechnology Information database (http://www.ncbi.nlm.nih.gov/blast/) for similarity to known genes. The deduced amino acid sequence was analyzed using the simple modular architecture research tool (SMART) (http://smart.embl-heidelberg.de/) to predict conserved domains. The presence and location of the signal peptide and the cleavage sites in the amino acid sequence were predicted using the SignalP 4.0 server (http://www.cbs.dtu.dk/services/SignalP/). The multiple alignment of the Tgfbr1 proteins of Zhikong scallop and other species was performed using the ClustalW2 multiple alignment program (http://www.ebi.ac.uk/Tools/msa/clustalw2/) and the GeneDoc multiple alignment editor (http://www.nrbsc.org/gfx/genedoc/index.html). The three-dimensional orthology model of the scallop Tgfbr1 kinase domain was predicted by the protein homology/analogy recognition engine (Phyre) 2.0 (http://www.imperial.ac.uk/phyre2/) and analyzed using Swiss-Pdb Viewer 4.04 (http://spdbv.vital-it.ch/). The minimum evolution method in the MEGA 5.0 software (http://www.megasoftware.net/) was used to construct an unrooted phylogenetic tree based on the deduced amino acid sequence of TGF-β type I and II receptors from different species. Aligned sequences were bootstrapped 1000 times to derive the confidence value for the phylogenic analysis.

### SNP Scanning and Genotyping

The total RNA was extracted from the striated muscle of 10 scallops from the full-sib family and reverse transcribed as described above. The Tgfbr1-f3 and Tgfbr1-r3 primers were used to amplify a 1625 bp cDNA fragment of Zhikong scallop *Tgfbr1* in a 50-µL PCR reaction containing 1× Phusion HF buffer, 0.2 µM of each primer, 0.2 mM of each dNTP (Invitrogen), 2.5 µL cDNA template, and 1 U Phusion DNA polymerase (New England Biolabs, MA, USA). The amplification was performed as follows: 95°C for 5 min; 25 cycles of 95°C for 10 s, 60°C for 20 s and 72°C for 30 s; and a final extension at 72°C for 5 min. The PCR products were purified and sequenced by Bioasia Biotechnology.

To identify gene variants, the *Tgfbr1* cDNA sequences from the 10 scallops were aligned and compared using the ClustalW2 program. A C/T substitution at position 1815 (relative to ATG the translation start codon, SNP c. 1815C>T) was identified in the 3′ untranslated region (UTR). The SNP was then genotyped in each of the 48 scallops from the full-sib family and 196 scallops from a population using the previously described high-resolution melting (HRM) method [34], [35]. Genomic DNA was extracted from the adductor muscle according to the traditional phenol/chloroform extraction method [33], and the Tgfbr1-f4 and Tgfbr1-r4 primers were designed to amplify a 101 bp DNA fragment surrounding the SNP locus. The PCR was performed in a 10 µL reaction containing 1× PCR buffer, 1.5 mM of MgCl_2_, 0.5 U T*aq* DNA polymerase (TaKaRa), 0.2 mM of each dNTP (Invitrogen), 0.1 µM of forward primer, 0.5 µM of reverse primer, 1×LCGreen Plus (Idaho Technology, UT, USA) and 20 ng genomic DNA. The amplification was performed as follows: 95°C for 5 min; 60 cycles of 95°C for 40 s, 63°C for 40 s and 72°C for 40 s; and a final extension at 72°C for 5 min. The probe Tgfbr1-Pb (3 µM) was added to each successfully amplified PCR product, and the mixture was denatured at 95°C for 10 min and then slowly cooled to 40°C. HRM genotyping was immediately performed using a Light-Scanner instrument (Idaho Technology) with continuous melting curve acquisition (10 acquisitions per °C) over a 0.1°C/s ramp from 40 to 95°C. The data were retrieved and analyzed using the Light Scanner software (Idaho Technology), followed by the manual curating of the genotype results.

### Quantitative Expression Analysis of Scallop *Tgfbr1*

The expression levels of scallop *Tgfbr1* at different developmental stages (fertilized egg, 4-cell stage embryo, blastula, gastrula, trochophore larva, D-shaped larva and veliger larva, n>500, 3 sets of samples for each stage) and in different adult tissues (mantle, gill, gonad, kidney, striated muscle and digestive gland) from six randomly selected scallops were analyzed using real-time quantitative reverse transcription PCR (qRT-PCR), according to Wang et al. [36], with three technical repeats for each PCR reaction. The primers Tgfbr1-f5 and Tgfbr1-r5 (Table 1) located in the exon 3 and exon 4, respectively, were used to amplify a 168 bp fragment of scallop *Tgfbr1* from cDNA template. The *β-actin* gene which is the commonly used reference gene in real-time qRT-PCR analysis for Zhikong scallop, was used as the internal control [30], [36]–[39]. Primers Actin-f and Actin-r (Table 1), were used to amplify 186 bp products of *β-actin*. The PCR products for both *Tgfbr1* and *β-actin* were purified and sequenced by Bioasia Biotechnology to verify the specificity of qRT-PCR. All of the amplification reactions were performed using a 7500 Real-Time System (Applied Biosystems, CA, USA) in a 20-µL volume containing 1× Real-time PCR Master Mix containing SYBR Green dye (TOYOBO, Osaka, Japan), 0.2 µM of each primer and 1 µL cDNA mix. The PCR amplification was performed as follows: initial denaturation at 95°C for 10 min, followed by 40 cycles of 95°C for 15 s and 60°C for 1 min. To confirm that only one product was amplified and detected, a dissociation analysis was performed at the end of each PCR by subjecting the samples to a constant decrease in temperature (from 95°C to 60°C). The results were analyzed using Real-time PCR Miner [40].

To compare the *Tgfbr1* expression levels in the striated muscles of scallops with different genotypes at the c. 1815C>T locus, six individuals of each genotype (CC, CT, and TT) were randomly selected. Total RNA was extracted from the striated muscle of the 18 scallops, and real-time qRT-PCR was performed as described above.

### Statistical Analysis

The Zhikong scallops from the full-sib family and the population were grouped according to their genotypes, respectively. The chi-squared test was used to examine Mendelian ratios in the family and Hardy–Weinberg equilibrium (HWE) in the population. For each growth trait, the mean value and standard deviation were calculated for each of the genotype group. A comparison of the means for each trait among different genotypes was performed one way ANOVA with post hoc test in both the family and population. In the family, for each genotype, six scallops were randomly selected, and the striated muscle weight and *Tgfbr1* expression level in the striated muscle of different genotypes were compared as described above. The comparison of expression levels of *Tgfbr1* among different developmental stages, and among adult tissues was performed as above, too. Pearson product-moment correlation was used to explore the relationships of *Tgfbr1* expression levels in striated muscle and the striated muscle weight. P values of less than 0.05 were considered statistically significant.

## Results

### Sequence Analysis of Scallop *Tgfbr1*

**Figure 1:**
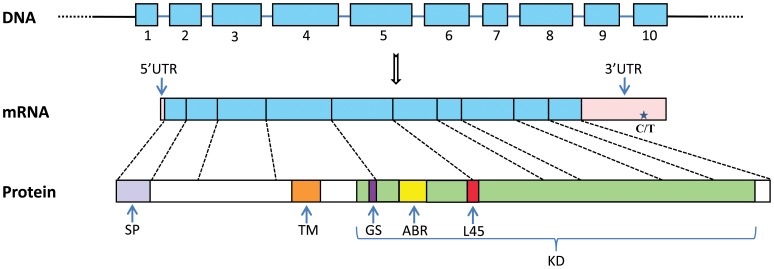
The structures of *Tgfbr1* gene and Tgfbr1 protein in Zhikong scallop. The gene contains 10 exons. The 3′ and 5′ UTR (untranslated region, pink), and exons encoding the amino acid sequences (blue) are shown relative to their lengths in the cDNA sequences obtained. The position of the SNP c. 1815 C>T in the 3′ UTR is indicated with a star. Protein domains are shown relative to their lengths and positions in the amino acid sequences. SP, signal peptide (gray); TM, transmembrane domain (orange); GS, SGSGSG motif (purple); ABR, ATP binding region (yellow); L45, L45 loop (red); KD, kinase domain (green).

A 1908 bp cDNA sequence was obtained by piecing together the 3′ and 5′ RACE sequences. It contains a 1575 bp open reading frame, a 15 bp 5′ UTR and a 318 bp 3′ UTR (Figure 1, Figure S1). A putative polyadenylation signal (ATTAAA) was identified at nucleotide positions 1714 to 1719. The translated amino acid sequence (524 aa) was most similar to that of Tgfbr1 and exhibited characteristic features of the vertebrate orthologs. The 29 amino acids at the amino terminus were predicted to be a cleavable signal peptide, and a potential transmembrane domain was located between amino acid residues 142 and 164. In the extracellular domain, 10 conserved cysteine residues were identified, which may participate in the formation of disulfide bridges, and three of these cysteines formed the characteristic CCX_5_C knot motif near the transmembrane region [41]. The intracellular region, containing the predicted serine/threonine kinase domain, was highly conserved. A glycine-serine (GS) box was found in the canonical SGSGSG motif, a specific site in TGF-β type I receptors that is phosphorylated by type II receptors [42]. The L45 loop (ADNKDNGTW) located at amino acid residues 283 to 291 perfectly matched the consensus motif of the vertebrate and oyster Tgfbr1 proteins [23], [43]; this motif determines the Smad specificity of TGF-β type I receptors [43].

**Figure 2:**
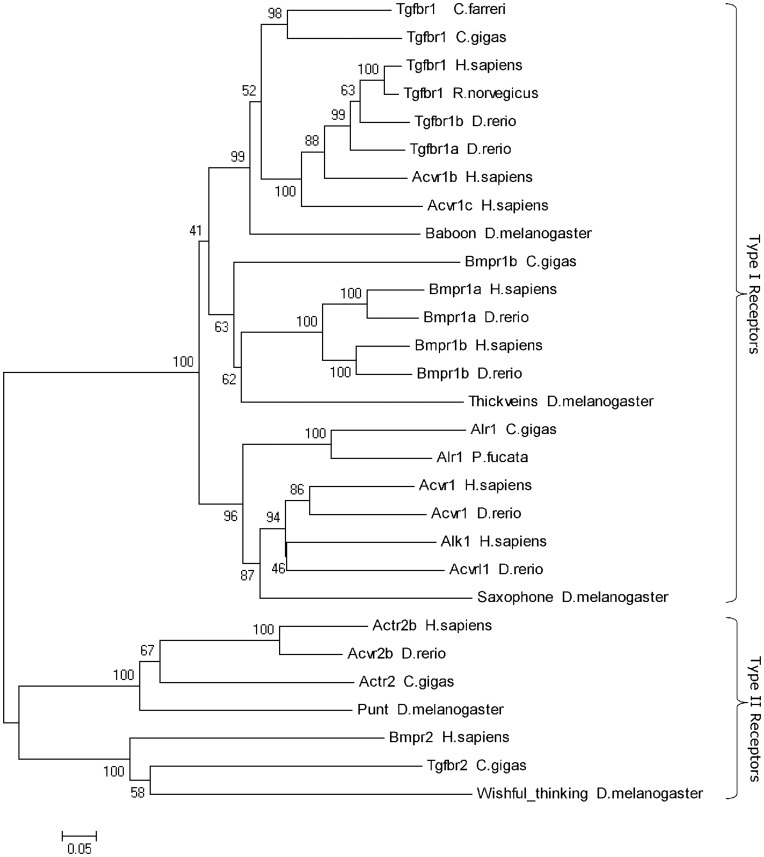
Phylogenetic analysis of scallop Tgfbr1 and other TGF-β superfamily receptors. This tree was built by the neighbor-joining method. Aligned sequences were bootstrapped 1000 times, and the numbers at the forks indicate the bootstrap proportions. The protein sequences used for phylogenetic analysis include the following: *Chlamys farreri* Tgfbr1 (JQ366030), *Crassostrea gigas* Tgfbr1 (CAD20573), *Homo sapiens* Tgfbr1 (CAF02096.2), *Rattus norvegicus* Tgfbr1 (AAA83216.1), *Danio rerio* Tgfbr1a (ABR20509.1), *D. rerio* Tgfbr1b (ABR20510.1), *H. sapiens* Acvr1b (AAH40531.1), *H. sapiens* Acvr1c (AAH22530.1), *Drosophila melanogaster* Baboon (AAF59011), *C. gigas* Bmpr1b (CAE11917), *H. sapiens* Bmpr1a (EAW80320.1), *D. rerio* Bmpr1a (AAI63471.1), *H. sapiens* Bmpr1b (AAH47773.1), *D. rerio* Bmpr1b (AAH81625.1), *D. melanogaster* Thickveins (XP_079689), *C. gigas* Alr1 (AJ309316), *Pinctada fucata* Alr1 (ADD80738.1), *H. sapiens* Acvr1 (AAH33867.1), *D. rerio* Acvr1 (AAI62317.1), *H. sapiens* Alk1 (CAA80255.1), *D. rerio* Acvrl1 (AAI00044), *D. melanogaster* Saxophone (AAA28878), *H. sapiens* Acvr2b (AAH96245.1), *D. rerio* Acvr2b (AAI64219.1), *C. gigas* Actr2 (CAR92545.1), *D. melanogaster* Punt (AAC41566), *H. sapiens* Bmpr2 (AAH52985.1), *C. gigas* Tgfbr2 (CAD20574), *D. melanogaster* Wishful thinking (AAF47832.1).

**Figure 3:**
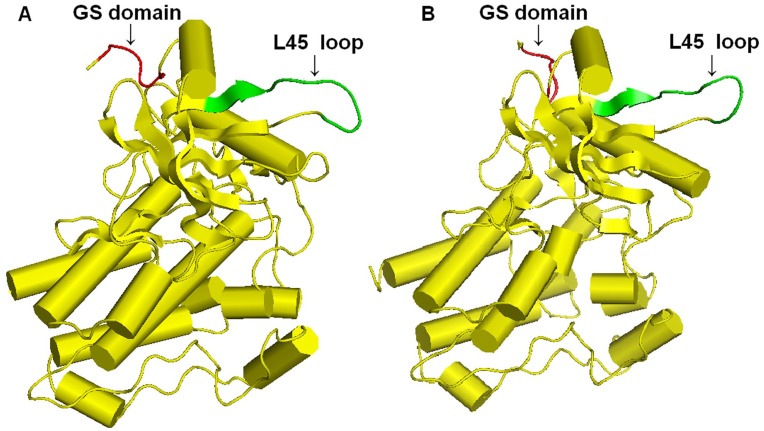
Molecular model of the three-dimensional structure of the kinase domain of scallop Tgfbr1 (A) and human Tgfbr1 (B). Cylinders and flat arrows represent α helices and β sheets, respectively. Conserved structures were found in the kinase domains of scallop Tgfbr1 and human Tgfbr1; the GS domain and L45 loop are colored red and green, respectively.

A phylogenetic analysis was performed to investigate the relationship of scallop Tgfbr1 with other type I and II receptors associated with ligands belonging to the TGF-β superfamily. A tree was generated by the alignment of 29 sequences from various phyla and showed a high degree of conservation between scallop Tgfbr1 and type I receptors in general but particularly with the TGF-β type I receptors (Figure 2). The three-dimensional model showed that scallop Tgfbr1 has a tertiary structure similar to human Tgfbr1 (PDB ID: 1B6C) [44] in the kinase domain (Figure 3) by which the receptor uses its L45 loop to interact with the L3 loop of Smad2 or Smad3 [6]. This result further suggested that this gene encodes the scallop ortholog of Tgfbr1 and that the possible intracellular messengers interacting with scallop Tgfbr1 might be Smad2 and Smad3. The scallop *Tgfbr1* gene and the deduced protein sequence have been deposited in the GenBank database under accession number JQ366030.

**Figure 4:**
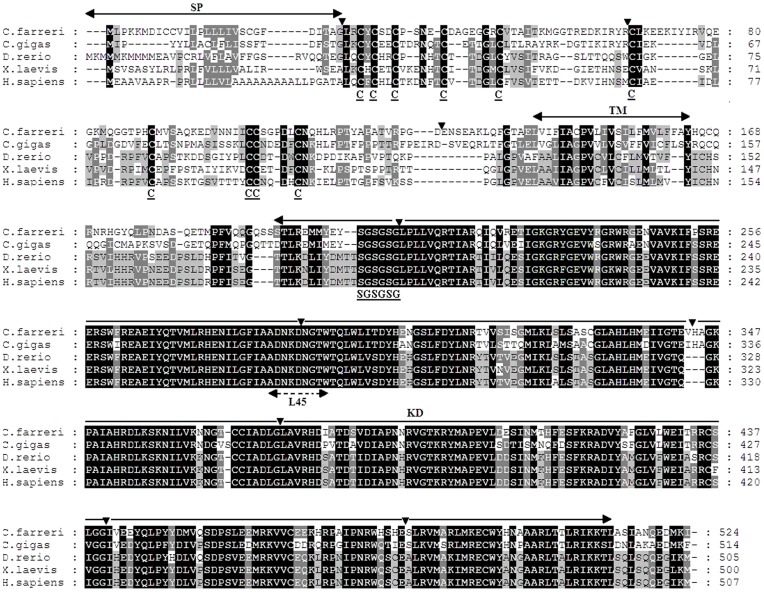
Multiple alignment of the scallop Tgfbr1 peptide sequence with orthologs from other species. Identical and similar amino acids are shaded. The signal peptide (SP), transmembrane domain (TM) and serine/threonine kinase domain (KD) are indicated with the solid-line horizontal arrows above the alignment. The ten conserved cysteine residues in the extracellular domain and the GS motif in the kinase domain are indicated with bold and underlined text. The L45 loop is highlighted with the dashed-line arrows under the alignment. The exon-intron junctions within the bivalve (scallop and oyster) *Tgfbr1* genes are indicated with black arrowheads. The species and the GenBank accession numbers are as follows: *C. gigas* (CAD20573), *D. rerio* (ABR20510.1), *X. laevis* (AAA84997.1), *R. norvegicus* (AAA83216.1) and *H. sapiens* (CAF02096.2).

A comparison of the protein sequence of scallop Tgfbr1 with those of Pacific oyster (*Crassostrea gigas*), zebrafish (*Danio rerio*), clawed frog (*Xenopus laevis*), rat (*Rattus norvegicus*), and human (*Homo sapiens*) revealed 63.8, 56.5, 57.2, 57.1, and 56.9% identity, respectively. In the serine/threonine kinase domain, the scallop Tgfbr1 exhibited 80.9 and 78.7% identity with the oyster and vertebrate orthologs, respectively, whereas the extracellular and transmembrane domains were much less well conserved (Figure 4). The cDNA sequence of scallop *Tgfbr1* was further analyzed by comparing it with the sequence database for the Zhikong scallop (∼50 × genome coverage; unpublished data). As with the Pacific oyster ortholog [23], the scallop *Tgfbr1* gene was organized into 10 exons (Figure 1, Figure S1), and the intron-exon boundaries and locations of the introns were conserved between Pacific oyster and Zhikong scallop (Figure 4). All of the intron-exon boundaries conformed to the GT-AG rule [45].

### Spatiotemporal Expression of Scallop *Tgfbr1*

**Figure 5:**
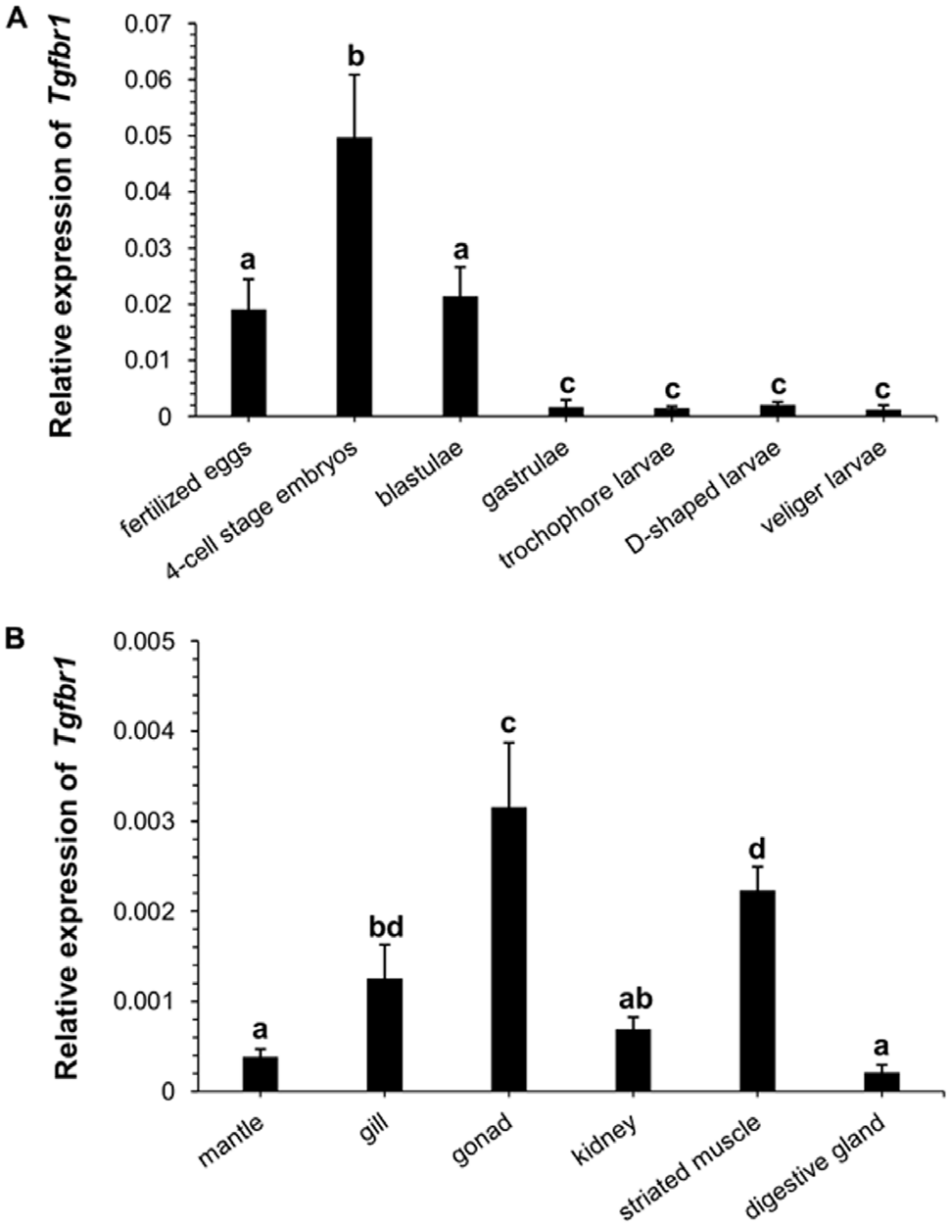
Spatiotemporal distribution of *Tgfbr1* transcripts in Zhikong scallop. (A) Relative expression levels of *Tgfbr1* at different embryonic and larval stages. Three biological replicates (n>500 for every replicate) were performed for each developmental stage, and three technical replicates were conducted for each PCR reaction. (B) Relative expression levels of *Tgfbr1* in various adult tissues (n = 6). The comparison of expression levels of *Tgfbr1* among different developmental stages, and among adult tissues was performed one way ANOVA with post hoc test. Bars with different superscripts indicate significant differences (P<0.05).

The expression of *Tgfbr1* in the embryos/larvae and adult tissues of Zhikong scallops was analyzed using real-time qRT-PCR. The amplification efficiency for the cDNA fragments of scallop *Tgfbr1* and *β-actin* was 0.998 and 0.997, respectively, according to the analysis by Real-time PCR Miner. Transcripts were detected in all of the developmental stages sampled, including fertilized eggs, 4-cell embryos, blastulae, gastrulae, trochophore larvae, D-shaped larvae, and veliger larvae (Figure 5A). *Tgfbr1* was expressed at significantly higher levels during the first 3 stages than the later larval stages, with the highest expression observed in the 4-cell embryos (significantly higher than in all the other stages). Sharp decrease in *Tgfbr1* expression was detected in the gastrulae, about 10 times lower compared to that in the blastulae stage, and the low level was kept during the later three larval stages sampled. No significant difference in *Tgfbr1* expression level was detected among the later four stages, or between fertilized eggs and blastulae. In the adult scallops, *Tgfbr1* was expressed in all of the sampled tissues (mantle, gill, gonad, kidney, striated muscle, and digestive gland), with higher expression levels observed in the gonad, and striated muscle, which is the main component of the scallop’s adductor muscle (Figure 5B). Significant differences in *Tgfbr1* expression were found between gonad and all the other tissues sampled. The digestive gland presented the lowest *Tgfbr1* expression, which showed significant difference with gill, gonad and striated muscle. No significant difference in *Tgfbr1* mRNA level was found among mantle, kidney and digestive gland, or between striated muscle and gill. Meanwhile, the data also showed that *Tgfbr1* expression levels in early developmental stages of embryos were one order of magnitude higher than in adult tissues of scallop (Figure 5).

**Table 2:** Growth traits of Zhikong scallops in a full-sib family with respect to genotypes at SNP c. 1815C>T in the *Tgfbr1* gene.

Genotype	N	GF	SL	SH	BW	STW	SMW*
CC	16	33.3	50.77±3.33^a^	55.74±3.37^a^	20.77±3.63	8.23±1.61	2.33±0.62^a^
CT	26	54.2	52.79±3.83^ab^	57.67±3.15^ab^	22.05±4.09	8.48±1.49	2.47±0.54^ab^
TT	6	12.5	54.79±2.47^b^	59.58±3.41^b^	24.12±2.23	9.25±1.21	3.02±0.47^b^

N, number of scallops; GF, genotype frequency (%); SL, shell length (mm); SH, shell height (mm); BW, body weight (g); STW, soft tissue weight (g); SMW, striated muscle weight (g). The growth traits are given as the mean ± standard deviation. The values with different superscripts within each column are significantly different (P<0.05). *, after Bonferroni correction, significant difference (p = 0.033) was detected between CC and TT genotype for SMW values.

### *Tgfbr1* SNP and Associations with Scallop Growth Traits

Only one SNP, located in the 3′ UTR of the gene (c. 1815C>T), was found in a comparison of the 1625 bp *Tgfbr1* cDNA sequences from 10 scallops (Figure 1). The allele frequency of C and T was 0.65 and 0.35, respectively. This SNP was further genotyped in each of the 48 scallops from the full-sib family using HRM assays. The frequencies of the CC, CT, and TT genotype were 33.3%, 54.2% and 12.5%, respectively (Table 2), conforming to Mendelian ratios (p = 0.105). Potential associations between the genotypes and each of the 5 growth traits (shell length, shell height, body weight, soft tissue weight, and striated muscle weight) were analyzed *via* one-way ANOVA with post hoc test. For all of the growth traits measured, higher values were detected in those individuals with the TT genotype than in those with either of the other two genotypes. The lowest values were detected in the scallops with the CC genotype, whereas the scallops with the CT genotype showed intermediate values. Significant differences were found between the scallops with the TT and CC genotypes for 3 of the 5 growth traits: shell length (p = 0.022), shell height (p = 0.017), and striated muscle weight (p = 0.011). After Bonferroni correction, significant difference was still detected in striated muscle weight between TT and CC genotype (p = 0.033). No significant difference was detected between individuals with genotypes TT and CT, or CC and CT for any of the growth traits tested (Table 2).

To further evaluate the association between SNP c. 1815C>T and growth traits, a Zhikong scallop population (196 individuals) was also studied. Similar as in the full-sib family, for all the measured growth traits, the highest values were detected in scallops with TT genotype, and lowest were in the CC genotype (Table 3). Significant difference was found in the values of striated muscle weight between scallops with genotype TT and CC (p = 0.037), and between TT and CT (p = 0.031). Chi-square test showed that the genotypic frequency at this locus was not in HWE (p = 0.017).

**Table 3:** Growth traits of Zhikong scallops in a population with respect to genotypes at SNP c. 1815C>T in the *Tgfbr1* gene.

Genotype	N	GF	SL	SH	BW	STW	SMW
CC	57	29.1	46.51±8.30	52.34±7.96	18.02±7.09	7.15±2.78	1.90±0.88^a^
CT	112	57.1	47.08±9.04	52.80±8.26	18.57±8.32	7.28±3.14	1.93±0.98^a^
TT	27	13.8	49.99±6.92	54.73±7.00	20.79±6.65	8.40±2.68	1.99±0.95^b^

N, number of scallops; GF, genotype frequency (%); SL, shell length (mm); SH, shell height (mm); BW, body weight (g); STW, soft tissue weight (g); SMW, striated muscle weight (g). The growth traits are given as the mean ± standard deviation. The values with different superscripts within each column are significantly different (P<0.05). Significant difference was detected in SMW values between genotype CC and TT (p = 0.037), and between CT and TT (P = 0.031).

### Association of SNP c. 1815C>T with *Tgfbr1* Expression

**Figure 6:**
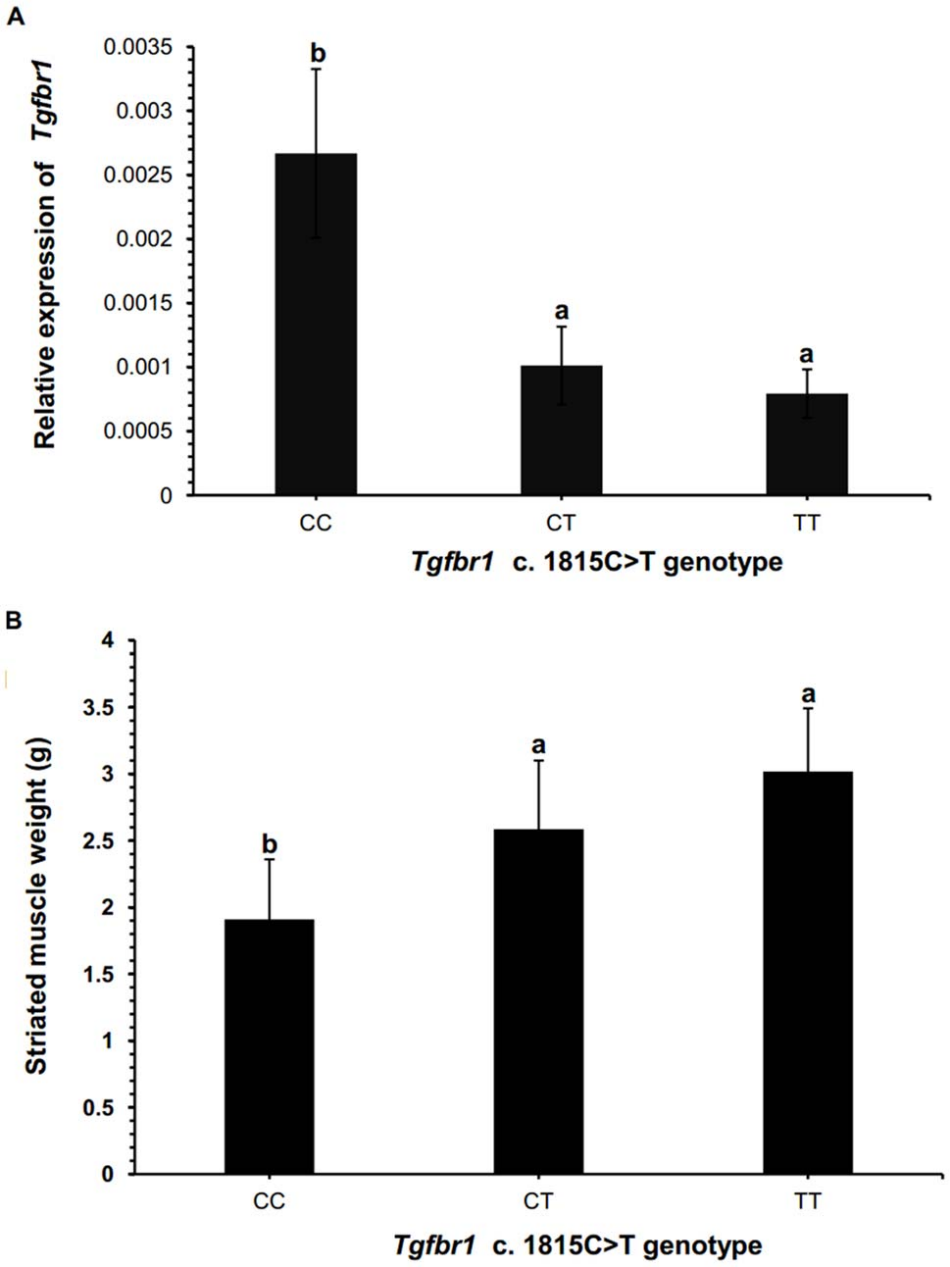
*Tgfbr1* expression levels in striated muscle and the striated muscle weights in scallops with different genotypes. For each genotype, six scallops were randomly selected. Associations of the SNP c. 1815C>T with the *Tgfbr1* expression level in striated muscle (A), and the striated muscle weight (B) were tested in the 18 scallops. One way ANOVA was performed with post hoc test to compare the expression levels of *Tgfbr1* among different genotypes, and the growth traits among the genotypes. Bars with different superscripts indicate significant differences (P<0.05). Pearson product-moment correlation analysis revealed inverse correlation between *Tgfbr1* expression level in striated muscle and the striated muscle weight (r = −0.56, p = 0.03).

Because *Tgfbr1* was highly expressed in striated muscle, and a significant difference was found between the striated muscle weights of the scallops with genotypes CC and TT at the c. 1815C>T locus, we speculated that this SNP might be correlated with *Tgfbr1* expression in this tissue. To test this hypothesis, for each genotype (CC, CT, and TT), we measured the *Tgfbr1* transcript levels in the striated muscles of six randomly selected scallops from the full-sib family using real-time qRT-PCR. The striated muscle weights of each genotype class were also compared. Significantly higher *Tgfbr1* expression levels were detected in the striated muscle of individuals with the CC genotype, as compared with individuals with genotype TT (p = 0.010) or CT (P = 0.019) (Figure 6A). The difference in *Tgfbr1* expression between CC and TT genotype was significant after Bonferroni correction (p = 0.030). The expression of *Tgfbr1* was the lowest in the scallops with the TT genotype, but it was not significantly different from the group with the CT genotype. Regarding the striated muscle weight in the same 18 scallops, significant differences were detected between genotypes CC and TT (p = 0.003; after Bonferroni correction, p = 0.009) (Figure 6B, Table S1, S2). Similar to the above association analysis performed with all the 48 individuals, the striated muscle weight was the lowest in the CC-genotype class and the highest in the TT class. These results showed that higher expression of *Tgfbr1* was in muscles of scallops with a lower muscle mass (and *vice versa*). Correlation analysis revealed that the expression level of scallop *Tgfbr1* in striated muscle was negatively correlated to striated muscle weight (r = −0.56, p = 0.030).

## Discussion

TGF-β signaling controls diverse cellular responses related to animal growth and development [3], [4], and the genes involved in this pathway have been extensively studied in economically valuable species, such as cattle, sheep, and pigs. These studies have provided helpful information, including gene sequences, expression profiles, gene function, allelic variations and associations, for the genetic improvement of these animals [17]–[19], [46]–[48]. For scallops, although genetic breeding aiming to improve production have been one of the main focuses of scallop farming, studies on the genetic regulation of scallop growth remain quite limited.

In this study, we cloned a TGF-β type I receptor gene, *Tgfbr1*, from the Zhikong scallop. Similar to its orthologs, scallop Tgfbr1 exhibited all of the characteristic features of type I receptor, such as the GS domain and the L45 loop (Figure 4). The conservation of the Tgfbr1 was further indicated by a comparison of the three-dimensional structure of the scallop Tgfbr1 kinase domain and the crystal structure of the kinase domain in human Tgfbr1 (Figure 3), which provides useful information for the gene function analysis, especially in respect to the activation of Tgfbr1 and Tgfbr1-Smad interactions in scallops. In addition to the sequence conservation, both oyster and scallop *Tgfbr1* contain 10 exons and 9 introns, and the intron-exon boundaries and locations of the introns are well conserved [23], indicating that the genomic structure and pre-mRNA splice sites of *Tgfbr1* are highly conserved in bivalves. In contrast, the mammalian *Tgfbr1* proteins, including human, bovine, mouse, and pig, all consist of 9 exons and 8 introns [46], [49]–[51].

*Tgfbr1* expression was detectable at all of the analyzed embryonic and larval stages of Zhikong scallop. Higher levels were detected in fertilized eggs, 4-cell embryos, and blastulae, suggesting that *Tgfbr1* may play an important role during early embryonic development in scallops; the high *Tgfbr1* expression in freshly fertilized eggs implies that the transcripts may be maternally derived. Lower but relatively stable expression of *Tgfbr1* was observed after the blastula stage, during early larval morphogenesis and metamorphosis, indicating a possible function for TGF-β signaling in the major organo-morphogenetic events that occur during scallop larval development, akin to the roles played by its vertebrate orthologs in body patterning [52], [53]. A similar profile of *Tgfbr1* mRNA expression was observed in oysters [23], with higher expression in early embryos and lower expression during the larval stages, also suggesting a similar function of Tgfbr1 in the development of the two bivalve species.

Among the sampled adult tissues of Zhikong scallops, the gonad and striated muscle presented the highest levels of *Tgfbr1* expression. Considering the high expression of *Tgfbr1* in newly fertilized Zhikong scallop embryos, its enrichment in the gonad might be indicative of high expression levels of *Tgfbr1* in oocytes and fertilized eggs. The expression of *Tgfbr1* in the gonad could also suggest that the gene might be involved in scallop reproduction. *Tgfbr1* has been shown to be critical for the structural integrity and function of the female reproductive tract, and genetic mutations in *Tgfbr1* can lead to fertility defects in women [54], In oysters, Corporeau et al. (2011) found that the inhibition of TGF-β function tends to reduce the gonadal area and that TGF-β most likely functions as an activator of germ cell development [55]. Future investigations of the *Tgfbr1* expression profile throughout the scallop reproductive cycle will yield more information on its potential role in gonad development and differentiation. The high level of *Tgfbr1* observed in striated muscle of Zhikong scallop implies the involvement of this gene in muscle growth and regulation. In mammals, TGF-β1, -β2, -β3, and myostatin, which signal *via* Tgfbr1, are all expressed in skeletal muscle [56]–[58] and participate in the regulation of muscle differentiation and proliferation [58]–[60]. Recent studies in fish showed that both Tgfbr1 and Tgfbr2 were highly expressed in skeletal muscle [61], and scallop *myostatin*, the only TGF-β pathway gene identified in scallop by far, was predominantly expressed in striated muscle [29], [30], reflecting a possibly role for TGF-β signaling in the aforementioned processes in aquatic species.

We identified a SNP (c. 1815C>T) in the 3′ UTR of scallop *Tgfbr1*. By genotyping the SNP in the offspring of a full-sib family, we found that the scallops with the TT genotype presented higher values for the 5 growth traits tested (shell height, shell length, body weight, soft tissue weight, and striated muscle weight) than the individuals with the CC or CT genotype (Table 2). Significantly higher values for shell height, shell length, and striated muscle weight were detected in the scallop with TT genotypes than those with CC genotypes, which implicates that the T allele might be related to the increase in shell and muscle growth, and the involvement of TGF-β signaling in the growth of these tissues in scallops. Previous studies of scallop traits showed that the shell height, shell length, and striated muscle weight were highly correlated [62], suggesting that the shell and striated muscle traits might be controlled by the same genetic regulatory mechanism. While after Bonferroni correction, significant difference was only detected in the striated muscle weight between TT and CC genotype, implying the close association of this locus with *Tgfbr1* expression regulation in striated muscle and the important role of *Tgfbr1* in striated muscle growth of scallop. This point was further suggested by the association detected in the scallop population where significant difference for trait values among genotypes of SNP c. 1815C>T was only found in the striated muscle weight, too, with higher values in TT type scallops than those with CC or CT genotype (Table 3).

At SNP c. 1815C>T, the genotypes of the scallop population were not in HWE, which mainly due to the low frequency of TT genotype (27 in 196). Meanwhile, in the full-sib family, the genotypes conformed to Mendelian ratios with relatively low p-value (0.105), which was also mainly caused by the lower TT genotype frequency (6 in 48) than expected (12 in 48). While the frequency of heterozygote CT was higher (112 in 196) than expected (96 in 196) in the population, implicating that the gene is under overdominant selection at this locus, and SNP c. 1815C>T might be functional.

To better understand the association between the SNP c. 1815C>T and striated muscle growth, we randomly selected six scallops from each genotype group in the full-sib family and analyzed the *Tgfbr1* expression levels in their striated muscle tissues. Significantly higher *Tgfbr1* transcript levels were detected in the striated muscle of the scallops with the CC genotype than in those with the TT or CT genotype, with lowest expression in TT type scallops (Figure 6A). We further investigated the association between the c. 1815C>T genotype and the growth trait in the same 18 scallops used for the above expression analysis; as expected, the individuals with the CC genotype had significantly lower values for the striated muscle weight than those with the TT or CT genotype (Figure 6B). These results, together with the inverse correlation of striated muscle weight and *Tgfbr1* expression, show that the C allele might be associated with high *Tgfbr1* expression in striated muscle and low striated muscle weight, whereas the T allele is associated with low level of Tgfbr1 expression and high striated muscle weight. In muscle cells, TGF-β signaling has been found to repress myogenesis by targeting critical myogenic regulators, e.g., myogenic regulatory factors (MRFs) and myocyte enhancer factor 2 (MEF2), and inhibiting muscle-specific gene expression [59]. Our data also imply that in scallops, TGF-β signaling may act through a similar mechanism as in mammals to repress muscle growth [4].

The SNP c. 1815C>T is located in the 3′ UTR of scallop *Tgfbr1*; thus, this locus might be related to post-transcriptional regulation at the mRNA level. It is also probably associated with the functional polymorphisms in other regulatory regions of scallop *Tgfbr1*, such as introns, 5′ regulatory region, or even the coding sequences. Further studies focusing on the SNPs across the entire genomic sequence of scallop *Tgfbr1* will be helpful to the identification of functional loci regulating *Tgfbr1* expression and scallop growth.

In conclusion, we cloned the first reported scallop TGF-β receptor gene, *Tgfbr1*, and analyzed its expression during early developmental stages and in adult tissues. We identified a SNP that is potentially associated with both scallop growth and *Tgfbr1* expression in striated muscle from the 3′ UTR of this gene. The associations are consistent with a function for TGF-β in negatively regulating cell growth. Furthermore, this study represents a first step to understand scallop growth through investigating the relationship between gene expression and growth traits with respect to SNP genotypes in the gene. This study will benefit our understanding in the genetic mechanism regulating scallop growth, and provides a candidate marker for the selective breeding of Zhikong scallop aimed at improving production.

## Supporting Information

Figure S1 **Nucleotide and deduced amino acid sequences of the** ***Tgfbr1*** **cDNA from the Zhikong scallop.**
(DOC)

Table S1 **Growth traits in 18 Zhikong scallops used for** ***Tgfbr1*** **expression comparison among genotype groups.**
(DOC)

Table S2 **Comparison of growth traits among genotype groups in 18 Zhikong scallops used for** ***Tgfbr1*** **expression comparison among genotypes.**
(DOC)
