# Correction: Molecular Characterization of TGF-β Type I Receptor Gene (*Tgfbr1*) in *Chlamys farreri*, and the Association of Allelic Variants with Growth Traits

**DOI:** 10.1371/annotation/75437eff-feb9-4592-baeb-ec7b98d1d212

**Published:** 2013-07-31

**Authors:** Huihui Guo, Zhenmin Bao, Jiqin Li, Shanshan Lian, Shi Wang, Yan He, Xiaoteng Fu, Lingling Zhang, Xiaoli Hu

There are multiple errors throughout the Funding section. The complete Funding section should read: "This work was supported by National Natural Science Foundation of China (30972239 and 31130054, http://www.nsfc.gov.cn/Portal0/default152.htm), National Basic Research Program of China (973 Program, 2010CB126406, http://www.most.gov.cn/), National High-Tech R&D Program (863 Program, 2012AA10A402, 2012AA092204, 2012AA10A401 and 2012AA10A405, http://www.most.gov.cn/), Program for New Century Excellent Talents in University (NCET-10-0716, http://www.moe.edu.cn/), and Natural Science Foundation of Shandong Province (ZR2009DM019, http://www.sdnsf.gov.cn/portal/). The funders had no role in study design, data collection and analysis, decision to publish, or preparation of the manuscript." 

