# Supplementary material for: Molecular Characterization of TGF-β Type I Receptor Gene (Tgfbr1) in Chlamys farreri, and the Association of Allelic Variants with Growth Traits
Source: PLoS One. 2012 Nov 29;7(11):e51005. doi: 10.1371/journal.pone.0051005 (PMC3510168; doi:10.1371/journal.pone.0051005)
Supplement: Figure S1 — Nucleotide and deduced amino acid sequences of the Tgfbr1 cDNA from the Zhikong scallop. (DOC) [file pone.0051005.s001.doc]

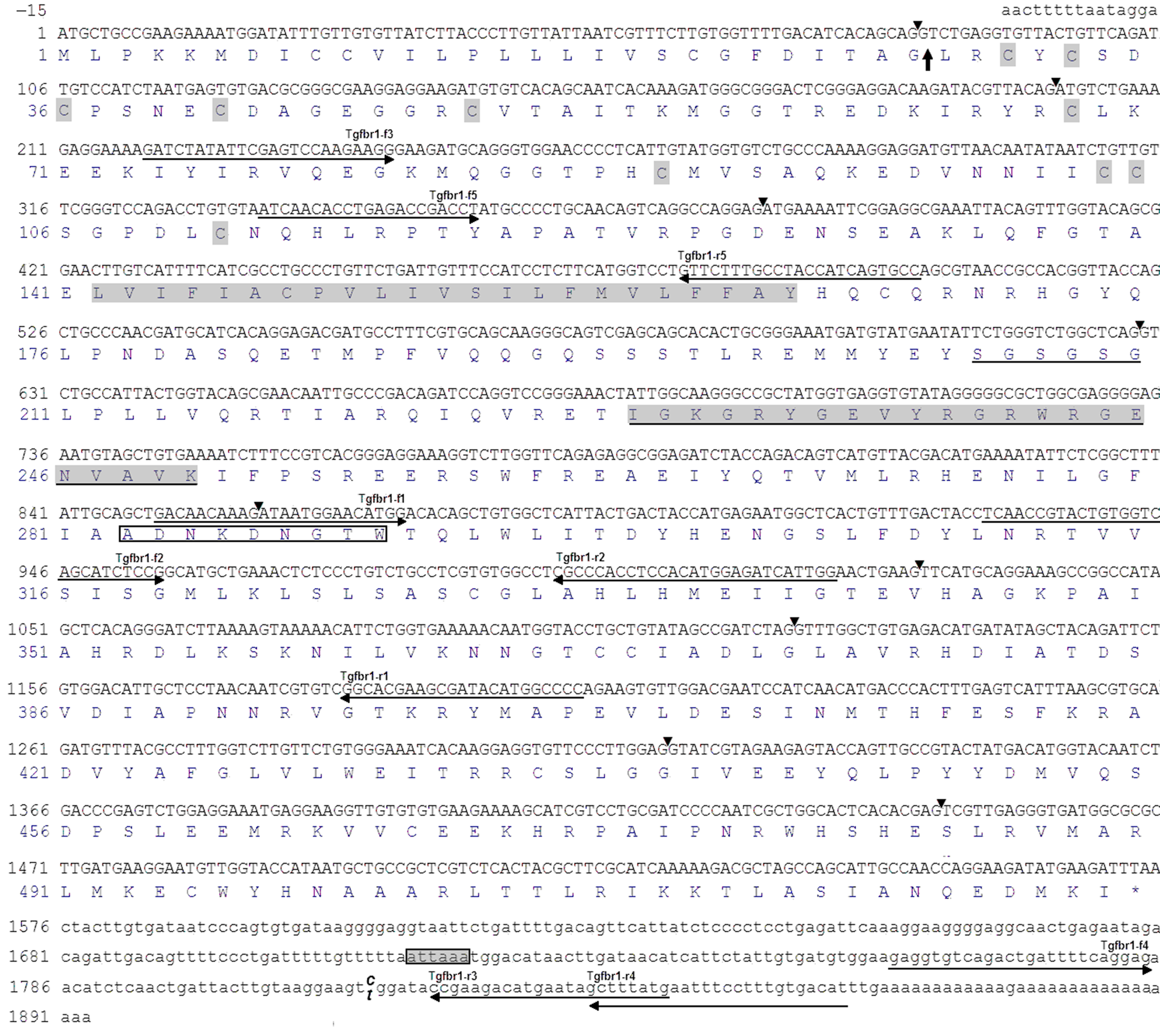


**Figure S1. Nucleotide and deduced amino acid sequences of the *Tgfbr1* cDNA from the Zhikong scallop.** The vertical arrow indicates the putative cleavage site of the signal peptide. The characteristic cysteine residues in the extracellular domain and the transmembrane region are shaded, and the characteristic type I GS box is underlined. A consensus ATP-binding region is both underlined and shaded. The receptor-Smad interaction L45 loop motif is boxed. The black arrowheads (vertical) mark the locations of introns. The putative polyadenylation signal is both boxed and shaded. The nucleotide at SNP c. 1815C>T is shown in bold italic. The locations and directions of the primers for scallop *Tgfbr1* in this study were indicated by horizontal arrows.
