# Supplementary material for: Molecular Characterization of TGF-β Type I Receptor Gene (Tgfbr1) in Chlamys farreri, and the Association of Allelic Variants with Growth Traits
Source: PLoS One. 2012 Nov 29;7(11):e51005. doi: 10.1371/journal.pone.0051005 (PMC3510168; doi:10.1371/journal.pone.0051005)
Supplement: Table S1 — Growth traits in 18 Zhikong scallops used for Tgfbr1 expression comparison among genotype groups. (DOC) [file pone.0051005.s002.doc]

**Table S1.** **Growth traits in 18 Zhikong scallops used for** ***Tgfbr1* expression comparison among genotype groups**

| Number | SL | SH | BW | STW | SMW | Genotype |
| --- | --- | --- | --- | --- | --- | --- |
| 1 | 53.4 | 58.48 | 23.09 | 9.4 | 2.34 | CC |
| 2 | 52.6 | 56.32 | 22.76 | 8.71 | 2.5 | CC |
| 3 | 47.91 | 53.39 | 16.98 | 6.99 | 2.01 | CC |
| 4 | 46.76 | 48.86 | 17.18 | 6.45 | 1.72 | CC |
| 5 | 48.45 | 53.93 | 19.61 | 8.95 | 1.44 | CC |
| 6 | 44.6 | 49.86 | 15.49 | 5.62 | 1.45 | CC |
| 7 | 50.24 | 56.09 | 17.93 | 6.55 | 2.06 | CT |
| 8 | 57.61 | 63.2 | 29.24 | 11.03 | 3.5 | CT |
| 9 | 54.31 | 58.53 | 22.24 | 7.54 | 2.41 | CT |
| 10 | 51.49 | 55.9 | 21.57 | 8.65 | 2.35 | CT |
| 11 | 51.46 | 56.07 | 21.44 | 9.19 | 2.34 | CT |
| 12 | 50.68 | 55.42 | 21.77 | 8.74 | 2.85 | CT |
| 13 | 58.06 | 64.84 | 27.24 | 10.01 | 3.27 | TT |
| 14 | 53.88 | 59.49 | 22.86 | 7.82 | 2.96 | TT |
| 15 | 54.83 | 59.62 | 24.61 | 9.62 | 3.27 | TT |
| 16 | 50.61 | 55.15 | 23.25 | 8.91 | 2.56 | TT |
| 17 | 55.68 | 56.88 | 20.99 | 8.09 | 2.4 | TT |
| 18 | 55.66 | 61.5 | 25.81 | 11.02 | 3.65 | TT |

SL, shell length (mm); SH, shell height (mm); BW, body weight (g); STW, soft tissue weight (g); SMW, striated muscle weight (g).
