# Supplementary material for: Molecular Characterization of TGF-β Type I Receptor Gene (Tgfbr1) in Chlamys farreri, and the Association of Allelic Variants with Growth Traits
Source: PLoS One. 2012 Nov 29;7(11):e51005. doi: 10.1371/journal.pone.0051005 (PMC3510168; doi:10.1371/journal.pone.0051005)
Supplement: Table S2 — Comparison of growth traits among genotype groups in 18 Zhikong scallops used for Tgfbr1 expression comparison among genotypes. (DOC) [file pone.0051005.s003.doc]

**Table S2. Comparison of growth traits among genotype groups in 18 Zhikong scallops used for *Tgfbr1* expression comparison among genotypes**

| Genotype | SL | SH | BW | STW | SMW* |
| --- | --- | --- | --- | --- | --- |
| CC | 50.02±3.97^a^ | 54.33±4.22^a^ | 20.06±3.18 | 7.78±1.51 | 1.98±0.49^a^ |
| CT | 51.57±3.24^ab^ | 56.68±3.35^ab^ | 21.45±4.31 | 8.52±1.61 | 2.52±0.57^ab^ |
| TT | 54.79±2.47^b^ | 59.58±3.41^b^ | 24.13±2.23 | 9.25±1.21 | 3.02±0.47^b^ |

SL, shell length (mm); SH, shell height (mm); BW, body weight (g); STW, soft tissue weight (g); SMW, striated muscle weight (g). The growth traits are given as the mean ± standard deviation. The values with different superscripts within each column are significantly different (P<0.05). *, after Bonferroni correction, significant difference (p=0.009) was detected between CC and TT genotype for SMW values.
